# Sutureless bioprosthesis may increase postoperative atrial fibrillation after aortic valve replacement

**DOI:** 10.1186/1749-8090-10-S1-A26

**Published:** 2015-12-16

**Authors:** Sameer D Thakur, Andrew Cheng, Hansraj R Bookun, David Lee, Chin Siew Lee, Xiao Bo Zhang, Shivanand Gangahanumaiah, Marco Moscarelli, Giacomo Bianchi, Pier A Farneti, Marco Solinas

**Affiliations:** 1 Department of Cardiothoracic Surgery, Royal Hobart Hospital, Australia; 2 Department of Cardiothoracic Surgery, University Hospital Geelong, Australia; 3 Department of Vascular Surgery, University Hospital Geelong; 4 Cardiology Research Unit, University Hospital Geelong, Australia; 5 Cardiothoracic Department, Fondazione Toscana G. Monasterio, Massa, Italy

## Background/Introduction

Benefits of sutureless aortic valve replacement (AVR) have been established. Most western centres have reported advantages in reduced cardiopulmonary bypass, cross clamp, ventilation, and postoperative recovery time. It has established a role in moderate to high-risk surgical patients requiring an AVR. However, the incidence of postoperative atrial fibrillation (POAF) in sutureless AVR is less known.

## Aims/Objectives

Investigate the incidence of POAF after sutureless AVR and compare that with the rate of POAF after sutured AVR.

## Method

From January 2001 to January 2015, 1417 AVR cases were performed. Demographic and perioperative data were collected prospectively. A total of 188 patients were excluded from analysis due to a preoperative history or incomplete data. We compared the rate of POAF after sutureless and sutured AVR cases overall and in subgroups divided by access (FS - Full Sternotomy; PS - Partial Sternotomy; MT - Mini-Thoracotomy). The incidence of POAF was identified by continuous cardiac monitoring.

Homogeneity of the sample was tested using multivariate regression and Kolmogorov-Smirnov tests, which did not identify any statistically significant confounding variables. Descriptive statistics were used to characterize samples with regards to demographic and perioperative variables.

## Results

A total of 1229 patients (604 females) were included in the analysis. The incidence of POAF in sutureless and sutured AVR cases was 35.8% and 29.5% respectively. The odds ratio for POAF is 1.33 (95% CI: 1.03-1.73; p = 0.031) with a sutureless valve. In subgroup analysis, POAF rates in the MT group for sutureless and sutured AVR were 33.1% and 22.0% respectively (OR 1.76 95%CI: 1.19 - 2.59; p = 0.004). POAF rates in the PS group for sutureless and sutured AVR were 50.9% and 33.3% respectively (OR 2.07 95%CI: 1.13-3.80; p = 0.019). FS had similar rates of POAF in both groups - sutureless 30.4% and sutured 32.3%.

## Discussion/Conclusion

Sutureless AVR is an important surgical option with proven advantages in moderate to high-risk patients. Prevention of POAF should be considered in patients whom a sutureless AVR is performed.

